# The effect of acute equine temporomandibular joint inflammation on response to rein-tension and kinematics

**DOI:** 10.3389/fvets.2023.1213423

**Published:** 2023-06-19

**Authors:** Nathalie A. Reisbig, Justin Pifko, Joel L. Lanovaz, Michael A. Weishaupt, James L. Carmalt

**Affiliations:** ^1^Department of Large Animal Clinical Sciences, Western College of Veterinary Medicine, University of Saskatchewan, Saskatoon, SK, Canada; ^2^College of Kinesiology, University of Saskatchewan, Saskatoon, SK, Canada; ^3^Equine Department, Vetsuisse Faculty, University of Zurich, Zurich, Switzerland

**Keywords:** rein-lameness, osteoarthritis, horse, TMJ, movement

## Abstract

**Background:**

Although the temporomandibular joint (TMJ) is the major contact point between the reins in the riders’ hand, the bit in the mouth, and the rest of the horse under saddle, the role of inflammation of this joint on equine locomotion and rein tension is unknown.

**Objective:**

To determine the effect of acute TMJ inflammation on rein-tension and horse movement when horses were long-reined on a treadmill.

**Study design:**

A randomized, controlled, cross-over design.

**Methods:**

Five horses were trained by one clinician to walk and trot on a treadmill wearing long-reining equipment instrumented with a rein-tension device and reflective optical tracking markers. Subjective assessment of horse’s dominant side, and movement, were determined without rein-tension (free walk and trot); and with rein-tension (long-reined walk and trot). Continuous rein-force data from both sides were collected over ~60s from each trial. Movement was recorded using a 12-camera optical motion capture system. One randomly assigned TMJ was subsequently injected with lipopolysaccharide and the treadmill tests repeated by investigators blinded to treatment side. A second, identical assessment was performed 10 days later with the opposite TMJ being the target of intervention.

**Results:**

All horses showed reduced rein-tension on the injected (inflamed) side. Increased rein-tension was required on the non-injected side at trot, to maintain them in the correct position on the treadmill post-injection. The only kinematic variable to show any significant change due to rein tension or TMJ inflammation during the walk or trot was an increase in forward head tilt in the presence of rein tension in the trot after injection.

**Main limitations:**

Low number of horses and investigation of response to acute inflammation only.

**Conclusion:**

TMJ inflammation changed, subjectively and objectively, the response to rein-input, but the horses did not become lame.

## 1. Introduction

Temporomandibular joint (TMJ) abnormalities are a common problem in humans and domestic animals. In humans, it is an umbrella term encompassing both intra-and extra-articular conditions and accounts for significantly higher health care costs and sick leave in affected compared to non-affected individuals (1). There have been numerous human studies on the neuromuscular effects of intra-oral splints, and reports detailing the correlation between different TMJ disorders and vertigo in elderly patients (2–4). Dental occlusion and TMJ abnormalities have also been reported to affect the center of gravity, gaze stability, physical performance, and fitness (5).

The inter-relationship between the TMJ and the rest of the body is of particular interest in equine performance, primarily because pain effects equine performance, whether or not it results in overt lameness (6). In addition, the reins and bit, by which the rider mainly communicates with the horse, can be a source of oral discomfort that changes the way a horse moves (7–9). Horses which are not necessarily classically lame but move differently when under saddle or tacked are known colloquially as “rein-” or “bridle-lame”; in German, the term “Zügel-lahmheit” is used. At times, this change can only be felt by the rider and is not appreciated by external observers. The effect makes accurate diagnosis extremely difficult. There have been several recent reports of TMJ osteoarthritis causing lameness and poor performance in sport horses (10, 11). There has been no research into the effect of TMJ inflammation on equine performance and motion, despite the fact that the TMJ is the conduit through which controlling rein pressure is exerted via the bit in the mouth to the rest of the horse under saddle.

Kinematic analysis and the use of instrumented reins represent objective methods of quantifying sub-clinical poor performance when used in combination with other diagnostic modalities such as perineural nerve or joint analgesia (12, 13). Using these methods, a significant amount of research has been published on rider impacts on equitation (14–18), the roles of tack (saddles, bits, and bridles) (19–22), and disease [specifically osteoarthritis (OA)] on movement of the horse (23–25).

The objectives of this study were 2-fold. Firstly, to evaluate the effect of acute inflammation of the TMJ using a lipo-polysaccharide (LPS) model on rein tension when horses were cued using long-reins at walk and trot. Secondly, to determine the effect of this inflammatory process on the 3D kinematics of horse movement at the same gaits.

The hypotheses were that; TMJ inflammation would affect the rein inputs necessary to control horse movement; and that this inflammation would change the 3D-kinematics of movement.

## 2. Materials and methods

### 2.1. Subjects

Five healthy horses, ranging in age from 2 to 25 years (Table 1) were loaned by owners who had completed a pre-approved institutional Animal Research Ethics Board informed consent form.

**Table 1:** Individual horse information.

Horse number	Breed	Sex	Age (years)	Dominant side	Walk speed (m/s)	Trot speed (m/s)
1	QH	F	9	L	1.5	3.5
2	QH	F	9	L	1.5	3.5
3	TB	F	25	L	1.4	3.5
4	TB	F	22	R	1.4	3.3
5	Clydesdale	MC	2	R	1.5	3.5

### 2.2. Protocol

Horses were initially trained to walk and trot, freely and while being long-reined, on an equine treadmill (Mustang 2200, Kagra AG, Fahrwangen, Switzerland). Optimal treadmill speeds were determined and recorded for each horse in free walk, free trot, long-reined walk, and long-reined trot (Table 1). The first author (NR) was responsible for controlling the horses on the treadmill during the entirety of the study (hereafter referred to as the handler) and remained blinded to treatment group throughout.

On day 1 of the trial, a snaffle-bit and bridle were fitted to the head of the horses. Long-reins were passed through the rings of a surcingle placed around the thorax, and attached to two wireless commercial rein-tension sensor systems, each weighing 79.7 gm (Ipos Technology, Eindhoven, The Netherlands) which were, in turn, attached to each side of the bit. A standard equine halter was placed on the head over the bridle-bit combination (ensuring that there was no interference with the reins and bit), which was used to guide the horses on the treadmill if needed.

**Figure 1:**
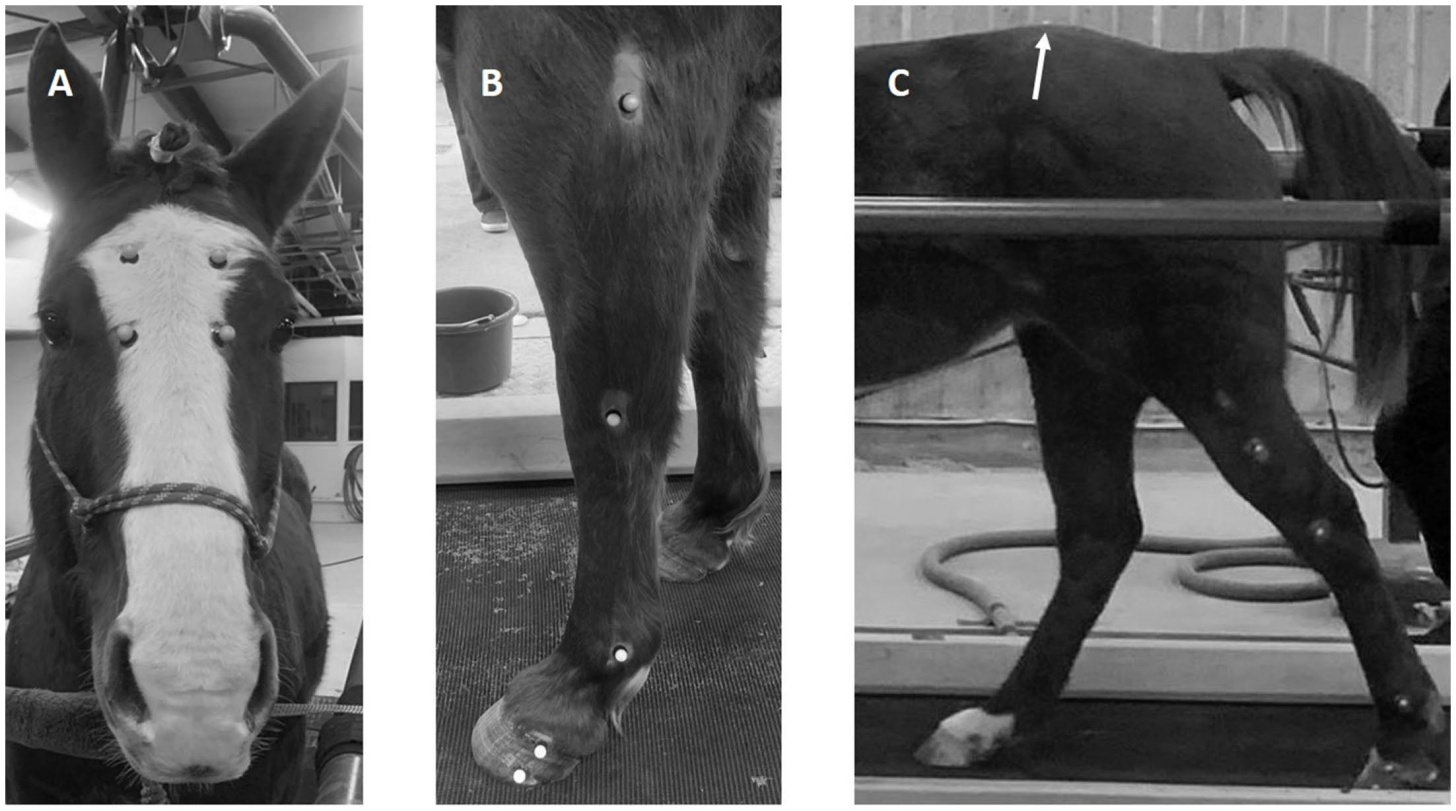
Photographs indicating the position of the reflective markers on the face **(A)**, the lateral aspect of the fore **(B)**, and hindlimb **(C)**. Note the position of the tuber sacrale marker on the dorsum in the midline **(C)**. One marker is not shown (withers).

Twenty-six reflective optical tracking markers (14 mm diameter sphere with a flat base) were attached to the horse using cyanoacrylate glue. Five on the lateral side of each limb (elbow/stifle, carpus/tarsus, fetlock, proximal, and distal hoof), one on the pelvic midline between the tuber sacrale, one on the dorsum at the withers, and four markers in a uniform rectangle on center of the face (Figure 1). The hair surrounding each location was shaved prior to marker attachment and the location of the marker base was traced on the skin using permanent marker. This allowed for the tracking markers to be replaced in the same locations for the session after the 10-day washout period.

All trials were performed on the equine treadmill (Mustang 2200, Kagra AG, Fahrwangen, Switzerland). Each trial started with the horse in a free walk (i.e., no tension on the reins), moving at its pre-determined comfortable speed, for approximately 1 min before switching to a free trot, again at the pre-determined speed, for approximately the same amount of time. The horse then repeated the walk and trot with tension applied to the reins and the horse was asked to “work into the bit” (26, 27) by the handler who was standing behind, and to the right of the horse, at the level of their hindquarters.

After the horses completed the treadmill trials, they were sedated with an intravenous injection of 200 mg xylazine hydrochloride (Rompun, Elanco Canada Ltd., 150 Research Lane, Guelph, ON, Canada) and the skin over both TMJs was clipped free of hair. These regions were then aseptically prepared using standard technique, and one randomly assigned discotemporal joint (the larger of the two joint compartments comprising the TMJ) received an intra-articular injection of 0.005 μg LPS (*E.coli* 055:B5, Sigma Aldrich Canada, ON, Canada) (28, 29). The senior author (JC) performed all the injections and was not responsible for handling the horse on the treadmill, or data collection. All other team members remained blinded to the side of injection.

Six hours after injection, the horses repeated the treadmill tests under the same conditions and horse-handling investigator. Afterward, the horses received 2 gm phenylbutazone (Phenylbutazone 20%, Rafter 8 Products, Calgary, AB, Canada) and were returned to their pen. Horses were observed for 48 h to ensure that there were no obvious negative sequelae to the intervention.

After a 10-day wash-out period (30), the entire procedure was repeated for each horse, with the other TMJ side receiving the injection.

### 2.3. Data collection

For the walk and trot trials with rein tension, the force in each rein was continuously measured using the rein-tension system as described above. The sensors recorded raw force data from each rein at a sample rate of 83.3 Hz, and data were stored on a mobile device and downloaded for post processing. For each walk or trot trial, approximately 60 s of continuous rein tension data were recorded.

Kinematic data were collected for all trials (with and without rein tension) using a 12-camera optical motion capture system at a sample rate of 200 Hz (Motive, OptiTrak, Corvallis, OR, United States). Continuous kinematic data were collected for 60 s for each walk and trot trial. For trials with rein tension, the kinematic data covered the same time period as the rein tension data.

### 2.4. Data analysis

**Figure 2:**
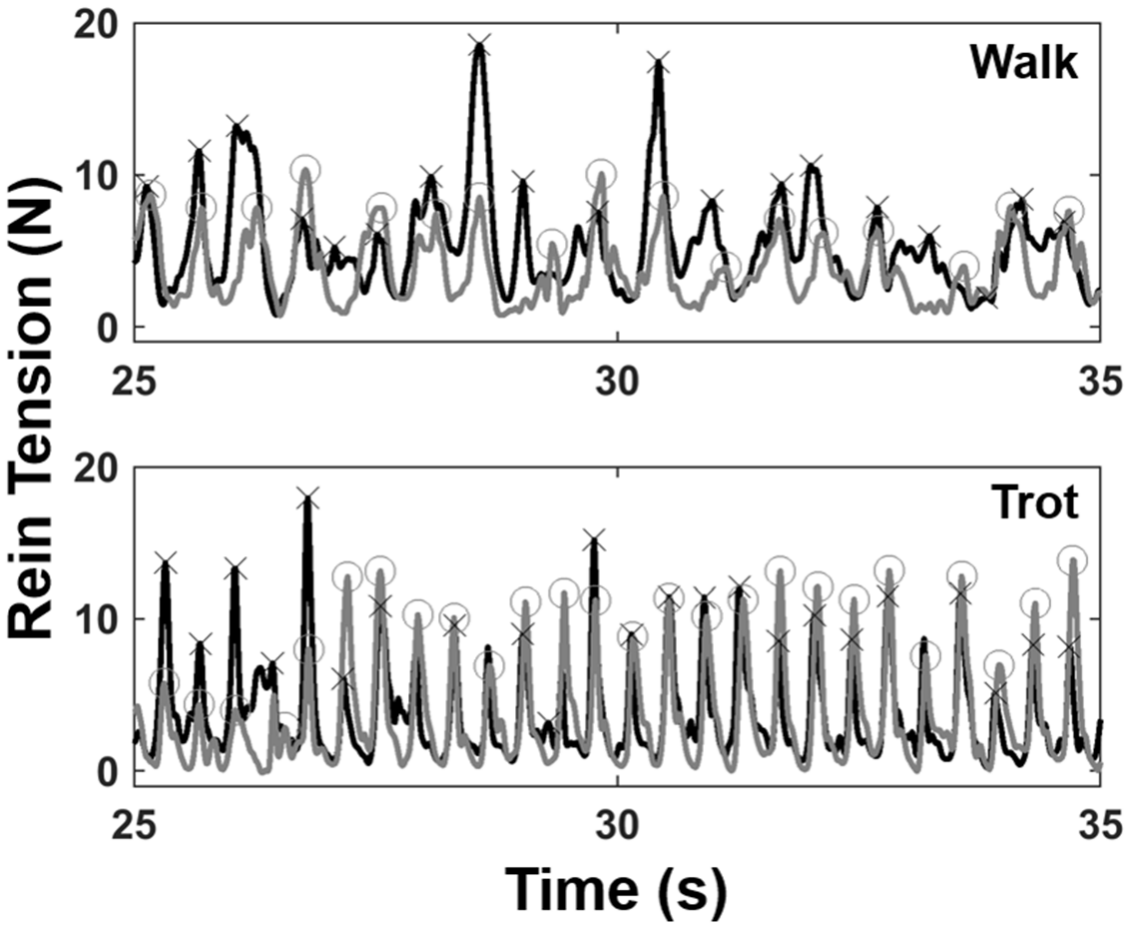
Exemplar rein tension data from 10 s of a walk (top) and a trot (bottom) trial. Left (gray) and right (black) force tracings are shown with force peaks identified (O = left peaks; X = right peaks).

The middle 50 s of rein tension data were extracted for each trial. Rein data were processed using custom software routines (Matlab 2019b, Mathworks, Natick, MA, United States). The data were first passed through a fourth order zero-lag low-pass Butterworth filter with a cutoff frequency of 20 Hz. Maximum force peaks were automatically extracted for each side (Figure 2). Individual force peaks were considered distinct if they occurred more than 300 ms apart in the walk or 200 ms apart for the trot. Left and right force peak pairs were then identified if they occurred within 100 ms of each other. For each left/right peak pair, a symmetry index (SI) was calculated based on the injected side such that SI = (injected side – non-injected side)/(injected side + non-injected side) and expressed as a percentage. Peak force data for each side and the SI were averaged across all strides for each trial of a given horse.

Kinematic data were initially processed using the motion capture software (Motive, OptiTrak, Corvallis, OR, United States) to ensure complete trajectories for all markers. The raw data were then exported and further processed using custom software routines (Matlab 2019b, Mathworks, Natick, MA, United States). The middle 50 s of kinematic data for each trial were extracted (approximately 70–90 strides) and low pass filtered at 15 Hz. Strides were automatically identified using the vertical velocity profiles of the right forelimb distal hoof marker and average stride time was calculated for each trial. Sagittal fetlock angles for each limb were calculated using the carpus/tarsus, fetlock and proximal hoof markers. Fetlock angle range of motion (ROM) was calculated for each stride and fetlock ROM SI were calculated for fore and hind limb pairs and averaged across all strides for each trial.

A three dimensional coordinate system for the head was established and tracked using the face markers with a medio-lateral axis going from right to left, a frontal “yaw” axis defined perpendicular to the surface of the face and the origin as the mean of the four face markers. The average angle of the head about the medio-lateral and frontal axes were calculated for each stride. The head medio-lateral angle was expressed such that a larger angle indicated the head was tilted more forward while the frontal axis angle was expressed such that zero was neutral and a positive angle indicated a tilt toward the injected side. The position of the head was expressed as the relative position between the origin of the head coordinate system and the location of the marker on the withers (i.e., withers location—head location). The average medio-lateral and vertical head position was calculated for each stride. For each trial, kinematic data were then averaged across strides.

### 2.5. Statistical analysis

For the rein tension peak forces, the effects of testing session (i.e., injection order), injection side and pre/post injection (time) were analyzed using a 2 × 2 × 2 (session × time × side) repeated measures ANOVA. For rein tension SI, a 2 × 2 (session × time) repeated measures ANOVA was utilized. For kinematics, data were collapsed across testing sessions as no main effects of session or interactions were found. Since kinematic data were collected with and without rein tension, the effects of the presence of rein tension and time were tested using a 2 × 2 (tension × time) repeated measures ANOVA for fore and hind fetlock ROM SI, mean head nod angle, tilt angle, head medio-lateral, and vertical position and stride time. Analyses were run for walk and trot data separately and alpha level was set to 0.05. All statistics were performed in SPSS (v.27, IBM).

## 3. Results

**Figure 3:**
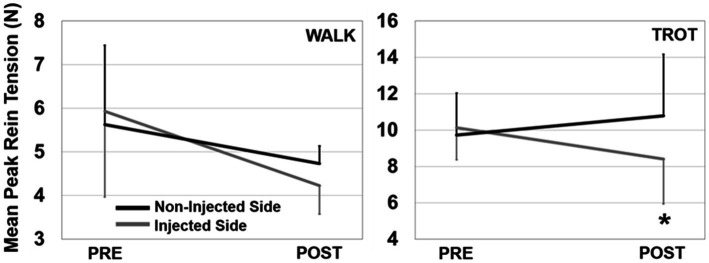
Peak rein tension for the walk (left) and trot (right) trials. Data are presented showing injected side × time interactions (error bars are SD). There was a significant interaction effect for the trot (shown with *) where the injected side had less tension than the non-injected side post injection.

**Figure 4:**
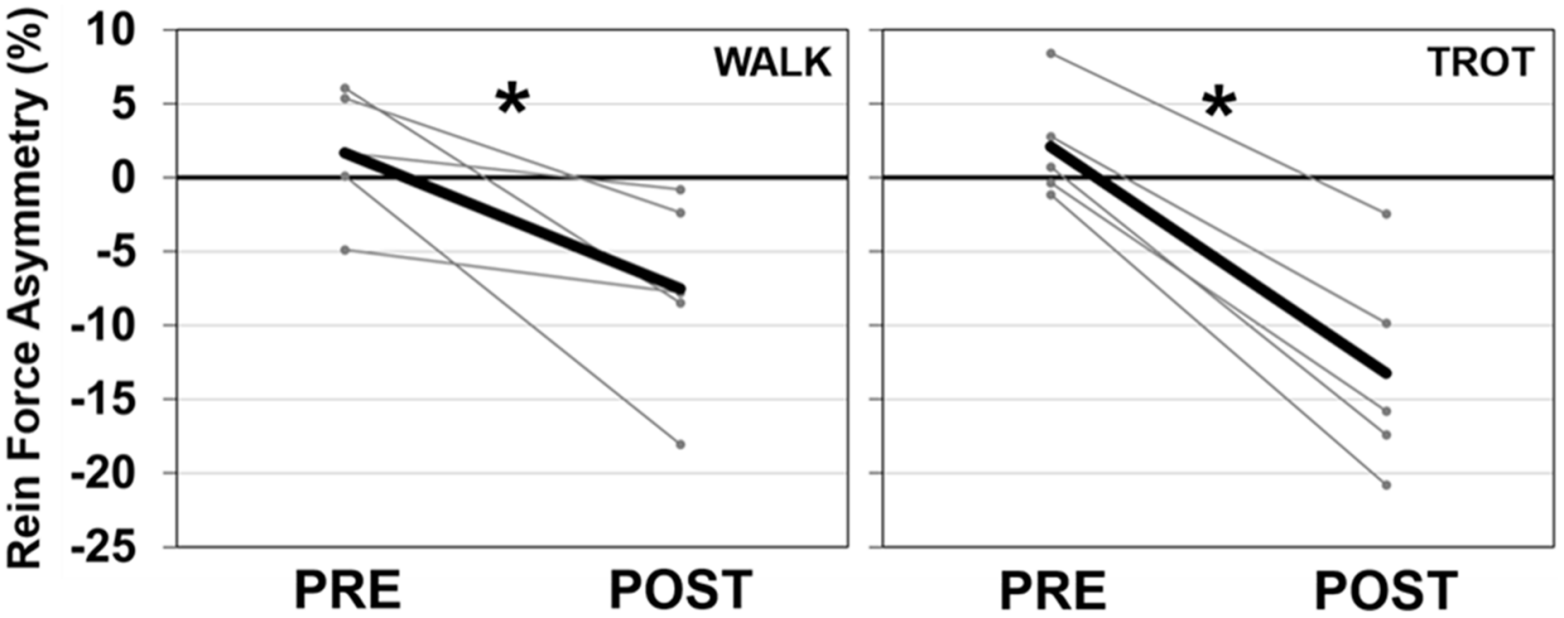
Peak rein tension SI for the walk (left) and trot (right) trials. Data for the mean (dark line) and individual horses (light lines) are presented. A negative SI percentage indicates less force on the injected side. There was a significant effect of time (shown with *) for both the walk and trot SI data.

There were no effects of injection order on peak rein forces at the walk (session 1: 5.6 N, session 2: 4.6 N, *p* = 0.335, η_p_^2^ = 0.23), while overall trot peak forces were slightly lower at the second injection time (session 1: 11.0 N, session 2: 8.5 N, *p* = 0.046, η_p_^2^ = 0.67). TMJ inflammation had no effect on average peak forces at the walk but there was a significant injection side × time interaction at the trot (*p* = 0.002, η_p_^2^ = 0.92) such that there was a decrease in peak force post-injection on the injected side (Figure 3). Asymmetry was significantly different after injection for both the walk (*p* = 0.043, η_p_^2^ = 0.68), and the trot (*p* = 0.001, η_p_^2^ = 0.96), with all horses showing a shift to reduce force (i.e., rein tension) on the inflamed side (Figure 4). There was no effect of injection order on asymmetry.

The only kinematic variable to show any significant change due to rein tension or TMJ inflammation during the walk or trot (Tables 2, 3) was forward head tilt which increased in the presence of rein tension in the trot (*p* = 0.042, η_p_^2^ = 0.68) after injection.

**Table 2:** Kinematic data for the walk (Mean and SD).

	No rein tension		With rein tension	
	PRE	POST	PRE	POST
Front fetlock ROM SI (%)	0.72 (1.81)	0.11 (1.64)	0.64 (1.21)	−0.45 (2.53)
Hind fetlock ROM SI (%)	2.26 (2.96)	0.05 (1.58)	0.96 (2.37)	0.16 (1.05)
Head forward tilt (deg)	71.61 (6.62)	72.99 (5.29)	79.71 (8.83)	79.22 (10.36)
Head frontal tilt (deg)	0.97 (5.62)	0.45 (2.71)	1.13 (1.98)	−1.11 (2.61)
Head ML position (cm)	−1.88 (6.43)	−1.29 (3.53)	−0.79 (2.55)	0.84 (2.97)
Head vertical position (cm)	22.36 (5.79)	21.4 (2.01)	19.22 (4.26)	20.37 (6.56)
Stride time (s)	1.21 (0.10)	1.22 (0.10)	1.20 (0.08)	1.20 (0.09)

ML, medio-lateral. Data are shown pre and post injection for trials with and without applied rein tension.

**Table 3:** Kinematic data for the trot (Mean and SD).

	No rein tension		With rein tension	
	PRE	POST	PRE	POST
Front fetlock ROM SI (%)	0.74 (0.80)	−0.78 (2.37)	0.63 (0.18)	−0.77 (2.19)
Hind fetlock ROM SI (%)	0.73 (1.48)	−0.24 (0.75)	0.84 (1.48)	−0.09 (0.71)
Head forward tilt (deg)	60.11 (3.45)	60.02 (4.77)	69.13 (13.13)	67.75 (12.01)^*^
Head frontal tilt (deg)	0.57 (3.15)	0.18 (4.01)	0.51 (2.00)	−0.26 (1.57)
Head ML position (cm)	0.27 (4.34)	−0.28 (5.25)	−0.66 (2.69)	0.81 (2.40)
Head vertical position (cm)	1.10 (4.19)	0.65 (3.28)	3.25 (10.41)	2.52 (9.42)
Stride time (s)	0.72 (0.05)	0.72 (0.05)	0.72 (0.05)	0.72 (0.05)

ML, medio-lateral; ^*^Significant difference between pre and post injection. Data are shown pre and post injection for trials with and without applied rein tension.

## 4. Discussion

The results of our study confirmed the first hypothesis, namely that rein inputs necessary to control horse movement would be affected by TMJ inflammation. The second hypothesis, that TMJ inflammation would change the horses’ locomotion, was not supported by the kinematic data.

The inter-relationship between mandibular position, TMJ inflammation, and the rest of the body is a complete unknown in the horse and a controversial topic, at best, in human orthodontics. Early work (31) showed that 70% of patients with TMJ osteoarthritis had improvements in balance, coordination and, or ataxia, after mandibular manipulation and this has been supported by a number of other researchers (2–4, 32, 33). This is contradicted by other reports in which patients with temporomandibular joint disorders, or abnormal disk position, did not have abnormal posture or muscular pain (34, 35).

Interestingly, there appears to be a role of dental occlusion and TMJ inflammation in physical fitness and human performance, which remains valid even at the elite level of human athletics (5, 36, 37). This inter-relationship is of particular interest to investigators involved in assessing equine performance because pain effects equine performance regardless of whether it results in overt lameness (6). This pain may manifest only when horses are under saddle, or tacked (rein-or bridle-lameness) as described above.

The findings of the current randomized, controlled, blinded study support previously published clinical papers linking TMJ abnormalities to poor performance in sport horses (10, 11). It shows that, at a trot, when rein tension was applied, the tension required to maintain the horse in the optimal treadmill position actually increased on the non-injected side. This makes sense from a control standpoint. The horse was worked into the bit (as evidenced by the significant increase in head tilt angle with rein tension) but was avoiding pressure on the injected side. To keep the horse straight on the treadmill, increased tension on the non-injected side was required.

The lack of statistical difference seen kinematically could be a function of only using five horses. Depending on the variable examined, a *post hoc* power calculation revealed that up to 63 horses would have been necessary to have been confident in a lack of statistical difference between pre-and post-injection movement. Subjectively, horses which had trotted in a stable, controlled, manner before TMJ injection became “unstable” when being “worked into the bit” by the hander after injection. In two (2/5) horses, seconds were being counted down toward the end of the data collection minute, because there was a concern that the horse would uncontrollably exit the treadmill by stepping on one of the side-rails. Interestingly, this observation was not corroborated by the objective kinematic data. It is possible that the handler controlled the instability sufficiently through the added rein-tension on the non-injected side to negate any objective differences. Had the investigators used a surcingle and side-reins at a set length (resulting in the same pressure being applied to each side of the bit), the kinematic results may have been different because there would have been no corrective input to any avoidance behavior displayed toward the injected side while the horse was at trot.

Finally, this study only investigated the role of acute inflammation. As osteoarthritis develops, the inflammatory process becomes chronic. When combined with the other classical manifestations of this chronic disease process (osteophytosis, synovitis, fibrosis, reduced range of motion, etc.), it is possible that the response to rein-input would be different and a secondary classic lameness may become apparent, similar to that noted in human patients (31). While this may seem implausible, chronic TMJ-OA in a sport horse was recently reported to effect an underlying baseline lameness (11); so a link between the two has already been established.

Determining that TMJ inflammation is causing poor performance, or rider-appreciated asymmetric rein-contact, is not easy. When presented with a suspected case it is important to have a logical, step-wise, approach to the evaluation. A detailed history, including a good understanding of the specific equestrian discipline, is critical before assessing the horse. Distant, complete physical, and lameness examinations should be performed; and only after excluding common problems (such as cardiac or musculoskeletal disease), should an investigation into the role of the TMJ be instigated. Options for assessment include injection of local analgesia, or diagnostic treatment of the joint(s); upper airway endoscopy; specialized tangential radiographic projections; computed tomography and, or, arthroscopic assessment of the offending joint (38). Overall, our experience has been that clinically significant TMJ pain in horses tends to occur in combination with other inter-related problems. All of these may require identification in order to successfully treat the patient. Despite this, the TMJ should not be excluded when considering the myriad causes of poor performance in the horse.

## 5. Conclusion

This study shows, under rigorous scientific conditions, that TMJ inflammation in the horse can be a source of rein contact avoidance. It elevates the importance of this enigmatic joint from relative obscurity—like neck and back pain 30 years ago—into one of serious contention when investigating nebulous equine performance concerns. Ultimately, more work is necessary, with a greater number of horses, to gain a better understanding of the relationship between inflammation of the equine TMJ and performance.

## Data availability statement

The raw data supporting the conclusions of this article will be made available by the authors, without undue reservation.

## Ethics statement

The animal study was reviewed and approved by the Institutional Animal Research Ethics Board and adhered to the Canadian Council on Animal Care guidelines for humane animal use (20200038). Written informed consent was obtained from the owners for the participation of their animals in this study.

## Author contributions

NR, JP, JL, MW, and JC contributed equally to study concept, design and execution of the trial, data interpretation, and manuscript preparation. All authors contributed to the article and approved the submitted version.

## Funding

This research was supported by the Mark and Pat Dumont Equine Research Fund at the University of Saskatchewan. The funding source had no role in study design, data collection, analysis or interpretation, in the writing of the manuscript, or in the decision to submit the manuscript for publication.

## Conflict of interest

The authors declare that the research was conducted in the absence of any commercial or financial relationships that could be construed as a potential conflict of interest.

## Publisher’s note

All claims expressed in this article are solely those of the authors and do not necessarily represent those of their affiliated organizations, or those of the publisher, the editors and the reviewers. Any product that may be evaluated in this article, or claim that may be made by its manufacturer, is not guaranteed or endorsed by the publisher.
